# Identifying Excessive Intake of Oil and Salt to Prevent and Control Hypertension: A Latent Class Analysis

**DOI:** 10.3389/fcvm.2022.782639

**Published:** 2022-04-06

**Authors:** Lu He, Yan Yan, Yuxiao Wang, Yudan Sun, Yuanyuan La, Jie Liu, Yutong Cai, Xi Cao, Qilong Feng

**Affiliations:** ^1^Department of Social Medicine, School of Public Health, Shanxi Medical University, Taiyuan, China; ^2^Department of Health Economics, School of Management, Shanxi Medical University, Taiyuan, China; ^3^First Hospital of Shanxi Medical University, Taiyuan, China; ^4^Department of Physiology, Key Laboratory of Cellular Physiology, Ministry of Education, Shanxi Medical University, Taiyuan, China

**Keywords:** hypertension, health behavior, latent class analysis (LCA), oil and salt in hypertension, public health

## Abstract

**Introduction:**

To identify health hazard behaviors and provide a basis for targeted management and intervention for patients with hypertension, we classified their health-related behaviors.

**Methods:**

A multi-stage random sampling method was used to conduct an on-site questionnaire survey among residents aged ≥15 years in a certain urban area of Taiyuan City, Shanxi Province, China. A latent class analysis was used to classify the lifestyle behaviors of patients with hypertension. The lifestyle behavior characteristics of different types of patients with hypertension and their awareness of hypertension were assessed.

**Results:**

The prevalence of hypertension in Taiyuan City was 19.5%. Patients with hypertension were classified into three clusters according to their lifestyle patterns: smoking and drinking (13.35%), excessive edible oil and salt intake (68.27%), and healthy behavior (18.38%). Comparing the three latent classes of lifestyle, the distribution of age, sex, marital status, and education level was different (*P* < 0.05). The awareness of hypertension and the rate of control among the three classes were also different (*P* < 0.05).

**Conclusion:**

The lifestyle behaviors of patients with hypertension have evident classification characteristics. Approximately two-thirds of the patients with hypertension have an excessive intake of oil and salt. Therefore, targeted and precise intervention measures should be taken to control the intake of oil and salt in this cohort.

## Introduction

Hypertension is one of the most important risk factors for stroke, heart disease, and kidney disease and is a major risk factor for premature death. Between 1990 and 2015, the loss of Disability Adjusted Life Year (DALY) associated with a systolic blood pressure of ≥140 mm Hg increased from 95.9 million (95% uncertainty interval, 87.0–104.9 million) to 143.0 million. It is estimated that approximately one-quarter to one-third of the world's adult population suffers from high blood pressure, and the number of patients suffering from it is 1 billion. This global increase was attributed to increasing age, Western eating habits, and obesity caused by sedentary and insufficient activity (1–4). In 2010, the total number of deaths in China caused by hypertension was 2.043 million, and 64.0% of all deaths caused by cardiovascular diseases were caused by hypertension (5). The ultimate goal of treatment strategies is to reduce the rates of cardiovascular mortality and morbidity related to chronic hypertension (6).

Hypertension is closely related to genetics and lifestyle behaviors. Although hypertension can be effectively controlled with drug therapy, another important means to reduce the risk of diseases can also be through the intervention of lifestyle behaviors (7–9). Regardless of the individual's genetic predisposition, adopting a healthy lifestyle can offset the genetic risk of high blood pressure and have a beneficial effect on reducing blood pressure and cardiovascular risk (10–12). A large number of studies have clearly confirmed that high sodium intake, a low potassium diet, being overweight, and obesity were important risk factors for hypertension in Chinese individuals (13, 14). With the yearly increase in the prevalence of hypertension, planning effective targeted measures for intervention in people with hypertension is a major problem in public health.

Many scholars have researched the precise intervention of hypertension in special populations from the perspective of lifestyle behaviors. For example, adopting a low-risk diet and lifestyle factors may prevent a large proportion of new-onset hypertension in young women (15). In middle-aged patients with hypertension, regular participation in physical activities will not only lower static blood pressure, but also weaken the response of blood pressure to all kinds of stressors (16, 17). Given the many influencing factors of lifestyle behaviors, whether the population with hypertension have certain category characteristics should be explored. This is an important breakthrough in controlling the prevalence of hypertension and reducing the hidden health hazards caused by hypertension. Effective targeted interventions should be adopted based on behavioral category characteristics.

There were a large number of traditional clustering research methods to explore the common characteristics of people with hypertension, however, these methods were suitable for continuous variables, such as general cluster analysis and factor analysis. Some studies pointed out that Latent Class Analysis (LCA) was suitable for discrete and binary classification, which is a more efficient way to explore healthy behaviors (18, 19). Latent class analysis is a data-driven analysis method that divides heterogeneous populations into homogeneous groups based on categorical indicator variables. A limited number of exclusive individual categories, classified as latent variables, are considered to constitute this class (20, 21). It can provide information on chronic disease intervention by classifying healthy behaviors and exploring the population's common characteristics.

Therefore, this study analyzed lifestyle behaviors related to hypertension to explore the different behavioral characteristics of the population based on the method of latent class analysis. The study also identified the key behavioral problems and focus groups of hypertensions. It could provide a scientific basis for precise intervention in the population with hypertension and effective prevention in healthy people.

## Materials and Methods

### Participants

The survey was conducted in 2019 in Taiyuan City, Shanxi Province, China. We found that 1% of the permanent population in an urban area was randomly selected using a multi-stage random sampling method. First, 13 streets were randomly selected from the 14 streets in the city's administrative divisions, from which 3–4 communities were randomly selected. Second, 100 households with two people in each household in each community were randomly selected. Finally, 6,823 permanent residents over 15 years of age were investigated, and 1,333 patients with hypertension were identified by testing their blood pressure three times on different days. All participants provided written informed consent. Ethics approval was granted by the Research Ethics Committee.

Inclusion criteria: Subjects who (1) were aged 35 and over; (2) were conscious, without cognitive impairment or mental illness; (3) were clearly informed of the purpose and significance of the study by the investigator; (4) were willing to cooperate and able to complete the survey; (5) had lived locally or were living locally for 6 months or more as of the date of investigation. The exclusion criteria included (1) those with critical diseases who were unable to complete the questionnaire; (2) obvious cognitive impairment or mental illness; (3) poor compliance and non-cooperation.

### Procedures

The patients were investigated uniformly by trained investigators using face-to-face questionnaire surveys. The content mainly included basic demographic factors (sex, age, educational level, marital status, family history, occupation), physical measurements (height, weight, blood pressure), health behaviors, awareness of hypertension, and occurrence of cardiovascular events (stroke, myocardial infarction, coronary heart disease). According to the “Guidelines for the Prevention and Treatment of Hypertension in China 2018,” patients with systolic blood pressure≥140 mm Hg and/or diastolic blood pressure≥90 mm Hg without taking antihypertensive drugs are considered hypertensive; systolic blood pressure is 140–159 mm Hg and/or diastolic blood pressure is 90–99 mm Hg is first-level hypertension; systolic blood pressure is 160–179 mm Hg and (or) diastolic blood pressure is 100–109 mm Hg is second-level hypertension; systolic blood pressure≥180 mm Hg and (or) diastolic blood pressure≥110 mm Hg is third-level hypertension. The awareness rate of hypertension was defined as the proportion of people who had been diagnosed with hypertension and knew that they had hypertension before the investigation. The treatment rate of hypertension was defined as the proportion of those diagnosed with hypertension and taking antihypertensive drugs in the past 2 weeks.

For daily oil and salt intake, we adopted the 3 day (including 1 Saturday or Sunday) 24-h dietary review method, combined with household weighing and bookkeeping to obtain the consumption of 3 day salt and oil in patient households. The sodium intake of all foods and condiments eaten by the patients in 3d was calculated according to the sodium content of various foods in the China food composition table 2002, and then converted into sodium chloride [(sodium intake/23) × 58.5] as salt intake (22, 23).

### Lifestyle Behaviors

According to previous studies, these behaviors were chosen as the explicit variables for model fitting, including smoking, drinking, intake of salt and oil, frequency of eating vegetables and fruits, exercise, and overweight or obesity. Considering that regular physical examination plays a key role in the early detection and prevention of cardiovascular disease, physical examination was included in the model fitting, with a total of nine variables.

**Figure 1:**
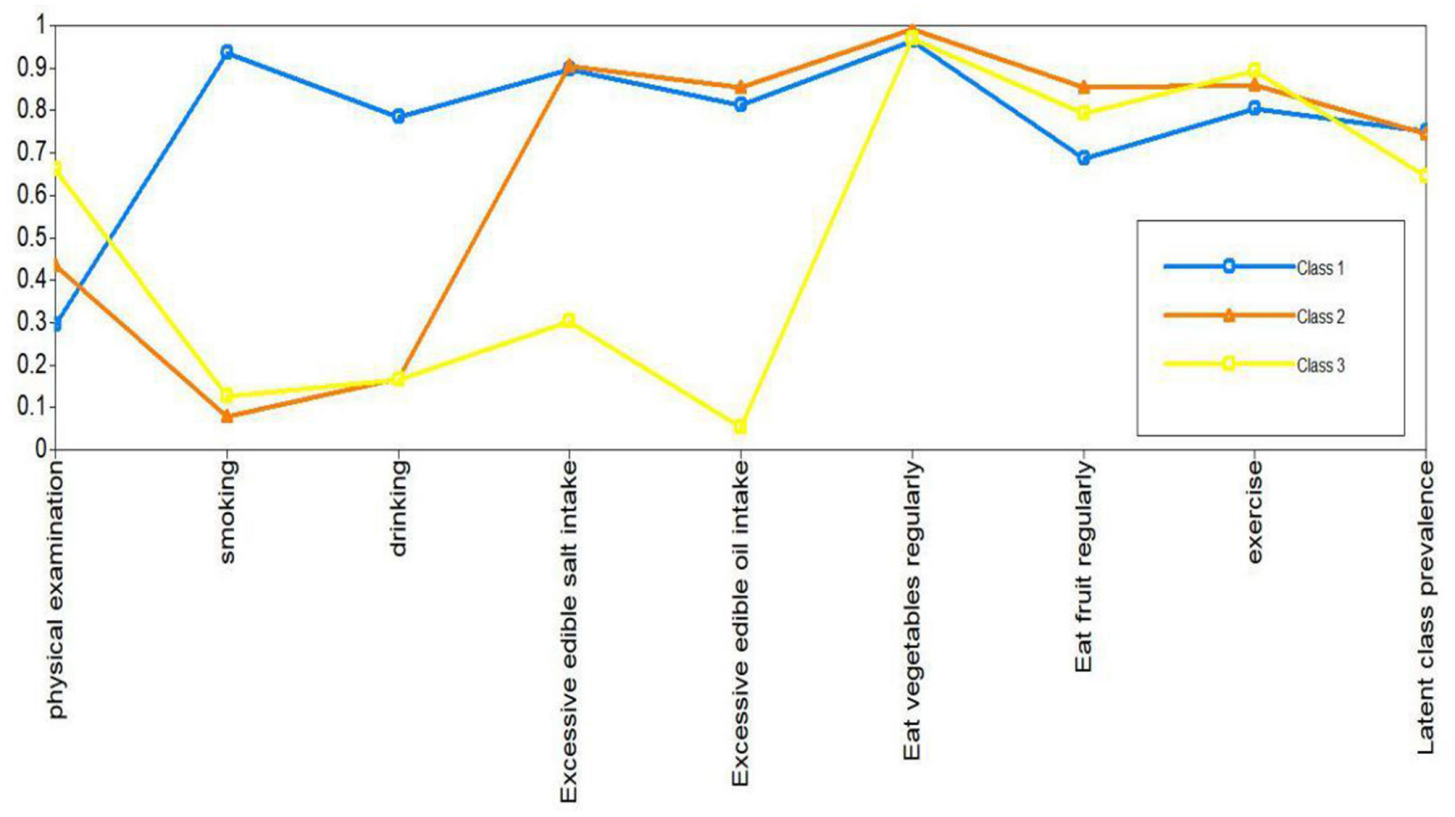
Study flowchart.

Smoking refers to smoking at least 1 cigarette per day and lasting for at least 1 year. Drinking refers to drinking at least once a week and drinking continuously or cumulatively for over 6 months. Participating in physical examination refers to having received a comprehensive physical examination in the past 2 years. The determination of excessive intake of edible salt and oil is based on the standards of the “Chinese Residents Dietary Guidelines 2016.” Adults consume >6 g of salt per day, and edible oil weighing >30 g is considered excessive. Participating in the physical exercise refers to doing jogging and brisk walking at least once a week and lasts for ≥10 min each time. Regular consumption of fruits and vegetables refers to eating fruits and vegetables ≥5 days a week. Overweight or obesity refers to body mass index (BMI) ≥24 kg/m (2). Awareness of hypertension is determined on whether self-reported hypertension and the question: “Have you been diagnosed with hypertension by your doctor?” Figure 1 shows our study flowchart.

### Data Analysis

Statistical analysis, including the chi-square test, multivariate logistic analysis, and multiple effect analysis, was performed using SPSS 25.0. The Mplus8.0 statistical software was used to analyze the behavior latent classes. The Pearson's test, likelihood ratio chi-square test, Akaike information criterion (AIC), and Bayesian information criterion (BIC) were information evaluation criteria. Among them, AIC and BIC are the most widely used indices in Latent Class Analysis (LCA) selection (24). They are based on the likelihood ratio chi-square test and can be used to compare models with different parameter restrictions. A smaller value represents better model fitness. Lin and Dayton (25) pointed out that the BIC index is more reliable when the sample size exceeds 1,000, otherwise, the AIC is better. If the significance of LMR is <0.05, it indicates that the k class solution is significantly better than the k-1 class solution. Furthermore, entropy was also used as an important criterion to choose the best model: it ranges from 0 to 1, with higher values indicating a better fit (23–25).

## Results

### Social and Demographic Characteristics of the Patients

Altogether, 6822 permanent residents aged ≥15 years were investigated. Among them, 1,333 patients with hypertension were detected, including 653 men (48.99%) and 680 women (51.01%). 876 (65.71%) had junior high school education or lower; 306 (23.0%) had high school/technical school/technical secondary school, and that of junior college education and bachelor's degree or higher were 7.1 and 4.2%), respectively. Seven-hundred and fifty-four cases (56.6%) aged 65 and above. One thousand and eighty-two cases (88.67%) were married, and 127 cases were divorced or widowed (9.52%).24 cases were unmarried (1.80%).

### Prevalence of Hypertension

The survey found 1,333 patients with hypertension, with a prevalence of 19.5%, of which 1,094 patients (82.07%) had first-level hypertension, 207 patients (15.53%) had second-level hypertension, and 32 had third-level hypertension. The awareness rate of hypertension was 53.38%, and the treatment rate was 47.71%. The prevalence of stroke and coronary heart disease was 6.45%, and that of myocardial infarction was 1.12%.

### Latent-Class Findings

We attempted to fit the latent class model on a scale from 1 to 5. For each potential category model, the G^2^ (2), AIC, and BIC were calculated. According to the BIC, when the number of categories is three, the BIC value is the smallest (BIC = 11701.363), and the LMR value is <0.05. Although entropy (0.771) is the most ideal when the category number is four, the LMR value is inferior when the number of categories is 3. The optimal model with three potential categories was chosen for a comprehensive consideration of various indicators (Table A1).

**Figure 2:**
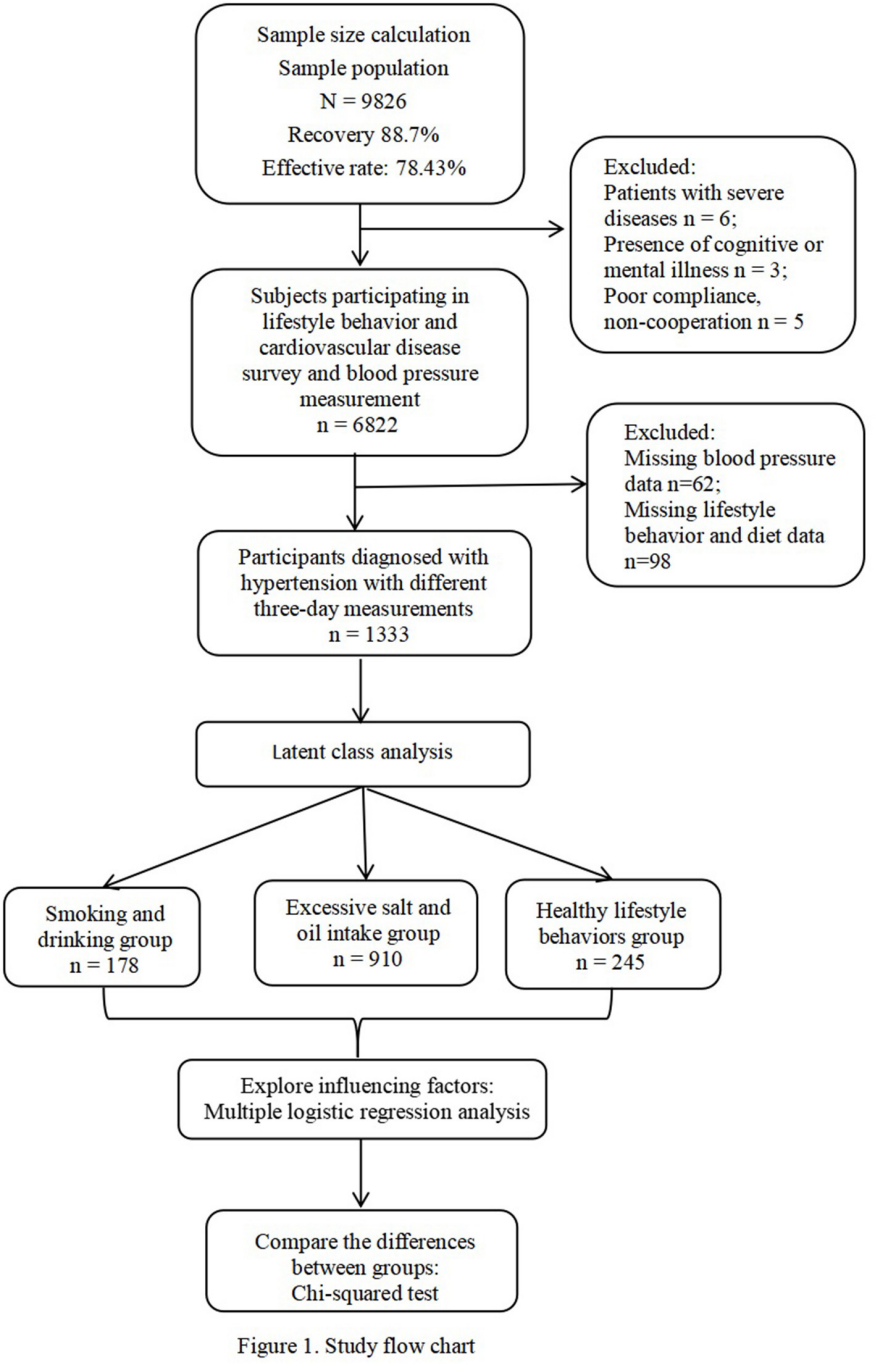
Latent class analysis profile pilot: the conditional probability distribution of nine lifestyle behavior.

Next, the patients with hypertension were classified according to the common characteristics of lifestyle behaviors in the three potential categories. Through parameter estimation, the conditional probability and potential category probability results of each of the nine items can be obtained. Class 2 had the largest proportion (68.2%), followed by Class 3 (18.4%) and Class 1 (13.4%). It can be seen from the conditional probability that the class 1 patients were more inclined to answer “Yes” on the questions on smoking or drinking, so class 1 was named the smoking and drinking group. The patients in class 2 tended to answer “Yes” on the question of whether their intake of edible oil and salt exceeded the standard, so they were named excessive edible oil and salt intake group. The class 3 patients in the nine items of lifestyle behaviors were more inclined to answer positive options, so they were named the healthy lifestyle behaviors group. There were 178, 910, and 245 people in the three classes, respectively. The prevalence of the patients in each latent class and item-response probability were shown in Figure 2.

### The Characteristics of the Three Latent Classes in Patients With Hypertension

The differences in the distribution of demographic factors such as age, sex, education level, and marital status of patients with hypertension in different potential classes were significant (*P* < 0.05). Among them, the smoking and drinking group were mainly men, aged 35~ years, and married people accounting for the largest proportion. In the excessive oil and salt group, women accounted for a larger proportion, and the patients aged ≥60 years old, and most of them were married, resigned or retired. The healthy lifestyle behavior group had the largest proportion of the people aged ≥60 years. The results are shown in Table 1.

**Table 1:** The characteristics of the three latent classes in patients with hypertension.

		**Smoking and**	**Excessive oil and**	**Healthy lifestyle**
**Characteristic**	**Group**	**drinking group (%)**	**salt intake group (%)**	**behaviors group (%)**	**χ^2^**	***P***
Age	≤ 35	7 (3.9)	41 (4.5)	12 (4.9)	74.992	**<0.001**
	36~	112 (62.9)	354 (38.9)	53 (21.6)		
	≥60	59 (33.1)	515 (56.6)	180 (73.5)		
Sex	Male	173 (97.2)	372 (40.9)	108 (44.1)	191.808	**<0.001**
	Female	5 (2.8)	538 (59.1)	137 (55.9)		
Educational level	Below junior high school	109 (61.2)	594 (65.3)	173 (70.6)	9.308	0.157
	High school	42 (23.6)	221 (24.3)	43 (17.6)		
	Junior college	17 (9.6)	62 (6.8)	16 (6.5)		
	Bachelor degree and above	10 (5.6)	33 (3.6)	13 (5.3)		
Marital status	Unmarried	2 (1.1)	16 (1.8)	6 (2.4)	62.570	**<0.001**
	married	170 (95.5)	828 (91.0)	184 (75.1)		
	Divorced/widowed	6 (3.4)	66 (7.3)	55 (22.4)		
Family history	No	110 (61.8)	534 (58.7)	139 (56.7)	1.094	0.578
	Yes	68 (38.2)	376 (41.3)	106 (43.3)		
Occupation	Working	89 (50)	239 (26.3)	50 (20.4)	47.781	**<0.001**
	Students	1 (0.6)	4 (0.4)	1 (0.4)		
	Retired/Unemployed	88 (49.4)	667 (73.3)	194 (79.2)		

Boldface indicates statistical significance *P < 0.05.

We then used multiple logistic to further explore the relationship between the demographic factors and potential classes (Table 2). Three lifestyle behavior classes were used as dependent variables, and the healthy lifestyle behaviors group was used as the control group. Every factor was used as an independent variable to be included in the multivariate logistic regression analysis. The results showed that male, 35~ year-old patients with hypertension were more likely to belong to the smoking and drinking group, while female, married, and 35~ year-old patients with hypertension were more likely to belong to the excessive edible oil and salt intake group. It can be seen that 35~ year-old patients with hypertension also have unhealthy lifestyle behaviors such as smoking, drinking, and intake of too much edible oil and salt.

**Table 2:** Multiple logistic regression analysis of potential classes of health behaviors.

**Classes**	**Characteristics**	**Group**	**B**	**SE**	**Waldχ^2^**	***P***	**OR (95%*CI*)**
Smoking and	Age	≤ 35	0.58	0.62	0.89	0.35	1.792 (0.53, 6.03)
drinking class		35~	1.62	0.27	35.83	**<0.001**	5.070 (2.98, 8.63)
excessive oil and		≥60	0b	.	.	.	.
salt intake group	Sex	Male	3.64	0.48	56.65	**<0.001**	38.079 (14.76, 98.24)
		Female	0b	.	.	.	.
	Age	≤ 35	0.09	0.43	0.05	0.83	1.10 (0.469, 2.57)
		35~	0.65	0.20	10.63	**<0.001**	1.921 (1.30, 2.84)
		≥60	0b	.	.	.	.
	Sex	Male	−0.36	0.16	4.96	**0.03**	0.70 (0.51, 0.96)
		Female	0b	.	.	.	.
	Marital status	Unmarried	0.64	0.61	1.09	0.30	1.90 (0.57, 6.31)
		married	1.252	0.22	32.83	**<0.001**	3.498 (2.28, 5.37)
		Divorced /widowed	0b	.	.	.	.

Boldface indicates statistical significance *P < 0.05.

### Awareness Rate and Control Rate of Hypertension in the Three Latent Class

The awareness rates of hypertension in the three potential classes were significant (*P* < 0.001), as shown in Table 3. The results showed that the awareness and control rates of the healthy lifestyle behaviors group were higher than those of both the smoking and drinking group and excessive edible oil and salt intake group.

**Table 3:** Comparison of three potential category classes.

**Classes**	**Number**	**Knowledge (%)**	**Control (%)**	**Apoplexy (%)**	**Myocardial infarction (%)**	**Coronary heart disease (%)**
Smoking and drinking group	178	46 (42.7)^*^	61 (34.3)^*^	8 (4.5)^*^	1 (0.6)	6(3.4)
Excessive edible salt and oil	910	483 (53.1)^*^	438 (48.1)^*^	54 (9.5)	12 (1.3)	64(7.0)
intake group
Healthy lifestyle behaviors group	245	152(62)	137(55.9)	24(5.8)	2 (0.8)	16(6.5)
χ^2^		8.809	19.570	6.074	0.542	3.311
*p*		**<0.001**	**<0.001**	**0.048**	0.783	0.191

Boldface indicates statistical significance ^*^P < 0.05.

### Prevalence of Cardiovascular Diseases in Three Latent Classes

The differences in the prevalence of stroke among the three potential classes were significant, although the differences in the prevalence of myocardial infarction and coronary heart disease among the three potential classes were not significant (Table 3). Moreover, the prevalence of stroke in the smoking and drinking group was higher than that in the healthy lifestyle behavior group, although the incidence rate of stroke in the oil and the salt excess group was lower than that in the healthy lifestyle behaviors group (*P* < 0.05).

## Discussion

This study found that the prevalence rate of hypertension in Taiyuan City was 19.5%, which was lower than that of China's 2012–2015 result of hypertension (27.9%) for residents aged ≥18 years. This may be related to the population's age composition and regional differences. The awareness rate of hypertension in Taiyuan City was 58.36%, slightly higher than in China (51.6%) in 2015. Patients with grade 1 mild hypertension accounted for the largest proportion of the patients with hypertension, so such patients should be the primary target of government intervention for chronic diseases. Through health promotion, one-and two-degree preventive measures may avert mild hypertension from transforming into severe hypertension and avoid the occurrence of cardiovascular events caused by hypertension (26). The study showed that there were evident classification features in lifestyle behaviors of patients with hypertension, which provided new evidence for behavioral interventions for patients with hypertension and chronic diseases. The study's finding was similar to those of Ghanbari and Jahangiry (21) who found significant differences in the risk behavior of patients with hypertension.

The innovation of this study lies in the application of the LCA in the analysis of hypertension population characteristics. Numerous studies have proved the importance of smoking, drinking, exercise, and other factors for the prevention and control of hypertension from the perspective of lifestyle by using traditional statistical methods. The clustering method was used to explore the life behavior characteristics of patients with hypertension. We found that after a series of health guidance measures, the effects of insufficient exercise, insufficient intake of fruits and vegetables, smoking and drinking on hypertension have decreased significantly. What should be of concern is the excessive consumption of oil and salt. This may bring new inspiration for the prevention and control of chronic diseases.

Through the Latent Class Analysis, from the perspective of lifestyle behaviors, we classified the patients with hypertension in the following groups: smoking and drinking (13.4%), excessive oil and salt intake (68.2%), and healthy lifestyle behaviors (18.4%). We compared the incidence of cardiovascular events among the three potential categories to understand the relationship between lifestyle behaviors and cardiovascular disease. Only the difference in the prevalence of stroke among the three potential classes was significant in this study, and epidemiological studies have shown the reliable relationship between dietary factors and cardiovascular morbidity and mortality (27, 28). Generally, the evidence of lifestyle having the potential role is very strong to prevent, treat, and even reverse the prevalence of cardiovascular diseases (29). Therefore, attention should be paid to intervene lifestyle in reducing the incidence of cardiovascular events and formulate appropriate intervention measures to reduce the incidence of complications in patients with hypertension. From the conditional probabilities of the nine items, it can be seen that all people with high blood pressure tend to respond positively with “yes” to sports and the frequent consumption of vegetables and fruits. This shows that people are increasingly aware of the importance of vegetables, fruits, and exercise in the prevention of chronic diseases. However, the study found that smoking and drinking remain important health hazard behaviors for people with high blood pressure, and it was found that men aged 35~ years were most likely to classify into the smoking and drinking group. Among the 6,823 people, the smoking rate was 17.73%, lower than that of Chinese adults (26.6%) in 2018 (29). Since 2003, various tobacco control policies formulated by the Chinese government have led to a decline in smoking rates, and publicity on the harms of tobacco and alcohol has also made the public aware of the health damage caused by smoking and drinking. The findings in this study suggested that although smoking and drinking played relatively less role in patients with hypertension, a series of policies were still needed for health promotion to reduce the health damage caused by poor smoking and drinking behaviors in patients with hypertension (30, 31).

Unexpectedly, the majority of the total patients belonged to the excessive edible oil and salt intake group. Our results also showed that married and middle-aged women were more likely to belong to this group. Given that the importance of married women in the development of the whole family's eating habits, it is crucial for interfering with women's cooking habits to prevent and control cardiovascular diseases. The daily salt intake in most countries is ~9–12 g, which is significantly higher than the World Health Organization's (WHO) recommended level of <5 g of salt per day (32). A research showed that the intake level of adult residents in China remains high, and the average salt intake was (9.6 ± 0.3) g/d (33). The WHO recommends that the daily intake of cooking oil for adults be 25–30 g. Cooking oil with a high saturated fat content is associated with a higher risk of developing cardiovascular disease (34). Our study found that while people pay attention to the benefits of eating fruits and vegetables for the prevention of chronic diseases, they ignore the more serious harm caused by excessive intake of salt and fat in the diet for cardiovascular disease.

Excessive oil and salt intake may be related to the unique eating habits of Chinese people. According to the 2021 Scientific Research Report of dietary guidelines for Chinese residents, dietary imbalance has become a major risk factor for the occurrence of chronic diseases, excessive oil, and salt intake is a common phenomenon in Chinese. Tan et al. (35) found that the median salt content of Chinese soy sauce is 4.4 times higher on average compared with similar British products (2015–2017). Only 13.4% of Chinese products meet the UK's 2017 salt target. Tian et al. (34) compared the current state of the Chinese adult diet with the deviation of the 2016 China Diet Pagoda (CFP), and the results showed that the average consumption of edible oil and salt was obviously higher than the upper limit of CFP 2016 (both *P* < 0.05). In particular, more than 50% of people overconsume cooking oil and salt.

Epidemiological studies have shown that there was a reliable relationship between dietary factors and cardiovascular morbidity and mortality (36). Intervention studies for healthy or high-risk populations have confirmed that diet control can improve modifiable risk factors for cardiovascular disease (37, 38) and reduce the incidence of cardiovascular events (39, 40). In the study of diet patterns, the Diet Method to Stop High Blood Pressure (DASH) model based on foods with low saturated fat content, polyunsaturated fat content, and low cholesterol content were recognized by the health organization as an effective diet for blood pressure control (41). Moreover, the dietary habits of Mediterranean countries have also been proven to extend their lives and prevent cardiovascular disease. This eating habit commonly includes regular consumption of fruits, vegetables, fish, beans, and the use of olive oil as cooking oil (42). Nutraceuticals is also seen as an unconventional method for treatment of dyslipidemia. Nutraceuticals and functional food ingredients that are beneficial to vascular health may represent useful compounds that are able to reduce overall cardiovascular risks induced by dyslipidaemia through parallel actions to statins or as adjuvants in case of failure or in situations where statins cannot be used (43). For instance, Izzo et al. found amelioration in calculated Framingham Risk Score sin patients suffering from metabolic syndrome and undergoing nutraceutical administration (44). Furthermore, Gunathilake et al. demonstrated that a poly-phenol-rich fruit-based functional beverage was able to significantly lower liver cholesterol and total and non-HDL-cholesterol levels in spontaneously hypertensive rats fed a cholesterol-rich diet (45).

Therefore, advocating the traditional Mediterranean diet and DASH diet is an important breakthrough for patients with hypertension. This diet mode can meet the nutritional needs of hypertensive patients and meet the requirements for preventing cardiovascular disease (46). To promote the effectiveness of dietary interventions, it is feasible to establish a multidisciplinary team composed of interdisciplinary experts who implement healthy lifestyle interventions (47). The cooperation of general practitioners, dietitians, health managers, and other teams in the community can effectively guide residents on healthy diets (48). Smartphones and the Internet can also be used to provide digital dietary intervention measures to significantly improve the health of residents (49).

Finally, by comparing the awareness and control rates of hypertension patients in the three potential classes, we found that whether it is awareness or control, the behavioral health group was higher than the other two relatively unhealthy categories. The awareness and control rates reflected the degree of patients with hypertension, understanding of their disease, and their willingness to take active measures to cope with hypertension, which was of great significance for effective hypertension control (50). From 2014 to 2017, Lu and Lu et al. conducted a population screening project for adults aged 35–75 years in 31 provinces in China, which showed that among the 1,738,886 people who participated in the survey, the awareness and control of hypertension rates were 36.0 and 5.7%, respectively (51). Understanding hypertension prevalence was conducive to the management and intervention of patients with a higher level of lifestyle behaviors. Therefore, there is an urgent need for comprehensive national strategies such as health education, free blood pressure screening, and improved access to affordable drugs to improve the prevention and control of hypertension in China (52). Focusing on people with unhealthy behaviors and taking them as the main subjects of hypertension-related knowledge publicity and intervention are recommended to improve the awareness and control rate of hypertension and reduce the risk of cardiovascular disease.

This study also had certain limitations. First, it was a cross-sectional study that only covered lifestyle behaviors of patients with hypertension during a certain period. We could not establish temporality. Reduction of edible salt and oil was often indicated in the treatment of certain chronic conditions such as hypertension and diabetes, but we were unable to account for potential dietary modifications made by these individuals due to the cross-sectional design of the study. Furthermore, our main measures of salt and oil were derived via 24 h dietary recall, which was subject to measurement error (27).To better verify the study's results, follow-up or cohort studies are required at a later stage. Another potential limitation was that dietary sodium intake was estimated by using 24-h recall. The best method to measure sodium intake in human subjects is 24-h urinary sodium excretion. However, it is not feasible to measure 24-h urinary sodium excretion with a large number of human subjects. Therefore, 24-h dietary recall and food-frequency questionnaire were the two methods considered to be the most cost-effective and feasible replacements for 24-h urinary sodium excretion for research done using a large number of human subjects. Even though these two methods have been used in previous human studies (22, 53, 54).

## Conclusion

The lifestyle behaviors of patients with hypertension have evident classification characteristics. Approximately two-thirds of the patients with hypertension have an excessive intake of oil and salt. Therefore, smoking, drinking, and an unhealthy diet, especially excessive intake of edible oil and salt, should be the current focus of interventions for people with hypertension, which provides a new idea for the implementation of an accurate and effective hypertension intervention strategy.

## Data Availability Statement

The original contributions presented in the study are included in the article/supplementary material, further inquiries can be directed to the corresponding author/s.

## Ethics Statement

The studies involving human participants were reviewed and approved by Shanxi Medical University Ethics Committee. Written informed consent to participate in this study was provided by the participants or their legal guardian/next of kin. Written informed consent was obtained from the individual(s), and minor(s)' legal guardian/next of kin, for the publication of any potentially identifiable images or data included in this article.

## Author Contributions

LH and YY conceived the idea. YW, YY, and YL participated in data collection and statistical analysis. JL drafted the manuscript. YC and XC gave many valuable comments on the draft and polished it. All authors have read and approved the manuscript.

## Funding

This study was supported by Shanxi Planning Philosophy and Social Sciences Project (2019B225).

## Conflict of Interest

The authors declare that the research was conducted in the absence of any commercial or financial relationships that could be construed as a potential conflict of interest.

## Publisher's Note

All claims expressed in this article are solely those of the authors and do not necessarily represent those of their affiliated organizations, or those of the publisher, the editors and the reviewers. Any product that may be evaluated in this article, or claim that may be made by its manufacturer, is not guaranteed or endorsed by the publisher.

## References

[1] Global burden of metabolic risk factors for chronic diseaseacollab-oration. Cardiovascular disease, chronic kidney disease, anddia-betesmortality burden of cardiometa bolic risk factors from 1980 to 2010:acomparatriver risk assessment. Lancet Diabetes Endocrinol. (2014) 2:634–47. 10.1016/S2213-8587(14)70102-0 24842598 PMC4572741

[2] GBD 2017 Risk Factor Collaborators. Global, regional, and national comparative risk assessment of 84 behavioural, environmental and occupational, and metabolic risks or clusters of risks for 195 countries and territories, 1990-2017: a systematic analysis for the Global Burden of Disease Study 2017. Lancet. (2018) 392:1923–94. 10.1016/S0140-6736(18)32225-6 30496105 PMC6227755

[3] Wang Y Peng X Nie X Chen L Weldon R Zhang W. Burden of hypertension in China over the past decades: systematic analysis of prevalence, treatment and control of hypertension. Eur J Prev Cardiol. (2016) 23:792–800. 10.1177/2047487315617105 26603746

[4] Shimbo D. Dietary and lifestyle factors in hypertension. J Hum Hypertens. (2016) 30:571–2. 10.1038/jhh.2016.57 27600029

[5] Wang Z Chen Z Zhang L Wang X Hao G Zhang Z. Status of hypertension in China: results from the china hypertension survey, 2012-2015. Circulation. (2018) 137:2344–56. 10.1161/CIRCULATIONAHA.117.032380 29449338

[6] Cuspidi C Tadic M Grassi G Mancia G. Treatment of hypertension: the ESH/ESC guidelines recommendations. Pharmacol Res. (2018) 128:315–21. 10.1016/j.phrs.2017.10.003 29080798

[7] Kjeldsen SE Aksnes TA Ruilope LM. Clinical implications of the 2013 ESH/ESC hypertension guidelines: targets, choice of therapy, and blood pressure monitoring. Drugs R D. (2014) 14:31–43. 10.1007/s40268-014-0049-5 24842751 PMC4070465

[8] Chobanian AV. Shattuck Lecture. The hypertension paradox–more uncontrolled disease despite improved therapy. N Engl J Med. (2009) 361:878–87. 10.1056/NEJMsa0903829 19710486

[9] Volpe M Gallo G Battistoni A Tocci G. Highlights of ESC/ESH 2018 guidelines on the management of hypertension: what every doctor should know. High Blood Press Cardiovasc Prev. (2019) 26:1–8. 10.1007/s40292-018-00297-y 30604199

[10] Ozemek C Tiwari S Sabbahi A Carbone S Lavie CJ. Impact of therapeutic lifestyle changes in resistant hypertension. Prog Cardiovasc Dis. (2020) 63:4–9. 10.1016/j.pcad.2019.11.012 31756356 PMC7257910

[11] Lantelme P. Hygiène de vie et hypertension artérielle chez la femme : spécificités féminines du traitement non medicamenteux [Lifestyle and hypertension in women, specific aspects of non-drug treatment]. Presse Med. (2019) 48(11 Pt 1):1257–60. 10.1016/j.lpm.2019.06.003 31307879

[12] Lim GB. Hypertension: lifestyle offsets genetic risk of hypertension. Nat Rev Cardiol. (2018) 15:196. 10.1038/nrcardio.2018.15 29493574

[13] Oliveras A de la Sierra A. Resistant hypertension: patient characteristics, risk factors, co-morbidities and outcomes. J Hum Hypertens. (2014) 28:213–7. 10.1038/jhh.2013.77 23985879

[14] Rantanen AT Korkeila JJA Löyttyniemi ES Saxén UKM Korhonen PE. Awareness of hypertension and depressive symptoms: a cross-sectional study in a primary care population. Scand J Prim Health Care. (2018) 36:323–8. 10.1080/02813432.2018.1499588 30139283 PMC6381520

[15] Forman JP Stampfer MJ Curhan GC. Diet and lifestyle risk factors associated with incident hypertension in women. JAMA. (2009) 302:401–11. 10.1001/jama.2009.1060 19622819 PMC2803081

[16] Palatini P. Cardiovascular effects of exercise in young hypertensives. Int J Sports Med. (2012) 33:683–90. 10.1055/s-0032-1304633 22562742

[17] Adamopoulos S Parissis J Kroupis C Georgiadis M Karatzas D Kara-volias G. Physical training reduces peripheral markers of inflammation in patients with chronic heart failure. Eur Heart J. (2001) 2:791–7. 10.1053/euhj.2000.2285 11350112

[18] Schreiber JB. Latent class analysis: an example for reporting results. Res Soc Adm Pharm. (2017) 13:1196–201. 10.1016/j.sapharm.2016.11.011 27955976

[19] Anderson LN Sandhu R Keown-Stoneman CDG De Rubeis V Borkhoff CM Carsley S. Latent class analysis of obesity-related characteristics and associations with body mass index among young children. Obes Sci Pract. (2020) 6:390–400. 10.1002/osp4.414 32874674 PMC7448165

[20] Lanza ST Collins LM Lemmon DR Schafer JL. PROC LCA: a SAS pro-cedure for latent class analysis. Struct Equ Model Multidiscip J. (2007) 14:671–94. 10.1080/10705510701575602 19953201 PMC2785099

[21] Ghanbari J Mohammadpoorasl A Jahangiry L Farhangi MA Amirzadeh J Ponnet K. Subgroups of lifestyle patterns among hypertension patients: a latent-class analysis. BMC Med Res Methodol. (2018) 18:127. 10.1186/s12874-018-0607-6 30419828 PMC6233281

[22] Song HJ Cho YG Lee HJ. Dietary sodium intake and prevalence of overweight in adults. Metabolism. (2013) 62:703–8. 10.1016/j.metabol.2012.11.009 23357528

[23] Elfassy T Mossavar-Rahmani Y Van Horn L Gellman M Sotres-Alvarez D Schneiderman N. Associations of sodium and potassium with obesity measures among diverse US hispanic/latino adults: results from the hispanic community health study/study of latinos. Obesity. (2018) 26:442–50. 10.1002/oby.22089 29318759 PMC5783725

[24] Kaufman L Rousseeuw PJ. Finding Groups in Data: An Introduction to Cluster Analysis. New York, NY: Wiley (2005).

[25] Hagenaars JA McCutcheon AL. Applied Latent Class Analysis. New York, NY: Cambridge University Press (2002).

[26] Lin TH Dayton CM. Model selection information criteria for non-nested latent class models. J Educ Behav Stat. (1997) 22:249–64. 10.3102/10769986022003249

[27] Lu J Lu Y Wang X Li X Linderman GC Wu C. Prevalence, awareness, treatment, and control of hypertension in China: data from 17 million adults in a population-based screening study (China PEACE Million Persons Project). Lancet. (2017) 390:2549–58. 10.1016/S0140-6736(17)32478-9 29102084

[28] Appel LJ Moore TJ Obarzanek E V ollmer WM Svetkey LP Sacks FM. A clinical trial of the effects of dietary patterns on blood pressure. N Engl J Med. (1997) 336:1117–24. 10.1056/NEJM199704173361601 9099655

[29] Kastorini C-M Milionis HJ Esposito K Giugliano D Goudevenos JA Panagiotakos DB. The effect of Mediterranean diet on metabolic syndrome and its components: a meta-analysis of 50 studies and 534,906 individuals. J Am Coll Cardiol. (2011) 57:1299–313. 10.1016/j.jacc.2010.09.073 21392646

[30] http://www.chinacdc.cn/jkzt/sthd_3844/slhd_4156/201908/t20190814_204616.html.

[31] Rhee M Na S Kim Y Lee M Kim H. Acute effects of cigarette smoking on arterial stiffness and blood pressure in male smokers with hypertension. Am J Hypertens. (2007) 20:637–41. 10.1016/j.amjhyper.2006.12.017 17531920

[32] Santo-Tomas M Lopez-Jimenez F Machado H Aldrich HR Lamas GA Lieberman EH. Effect of cigar smoking on endothelium-dependent brachial artery dilation in healthy young adults. Am Heart J. (2002) 143:83–6. 10.1067/mhj.2002.119765 11773916

[33] Rust P Ekmekcioglu C. Impact of salt intake on the pathogenesis and treatment of hypertension. Adv Exp Med Biol. (2017) 956:61–84. 10.1007/5584_2016_147 27757935

[34] Tian X Huang Y Wang H. Deviation of Chinese adults' diet from the Chinese food pagoda 2016 and its association with adiposity. Nutrients. (2017) 9:995. 10.3390/nu9090995 28885553 PMC5622755

[35] Tan M He FJ Ding J Li Y Zhang P MacGregor GA. Salt content of sauces in the UK and China: cross-sectional surveys. BMJ Open. (2019) 9:e025623. 10.1136/bmjopen-2018-025623 31548352 PMC6773338

[36] Mahmood S Shah KU Khan TM Nawaz S Rashid H Baqar SWA. Non-pharmacological management of hypertension: in the light of current research. Ir J Med. Sci. (2018) 188:437–52. 10.1007/s11845-018-1889-8 30136222

[37] Alissa EM Ferns GA. Dietary fruits and vegetables and cardiovascular diseases risk. Crit Rev Food Sci Nutr. (2017) 57:1950–62. 10.1080/10408398.2015.1040487 26192884

[38] Doughty KN Del Pilar NX Audette A Katz D. Lifestyle medicine and the management of cardiovascular disease. Curr Cardiol Rep. (2017) 19:116. 10.1007/s11886-017-0925-z 28980137

[39] Saneei P Salehi-Abargouei A Esmaillzadeh A Azadbakht L. Influence of dietary approaches to stop hypertension (DASH) diet on blood pressure: a systematic review and meta-analysis on randomized controlled trials. Nutr Metab Cardiovasc Dis. (2014) 24:1253–61. 10.1016/j.numecd.2014.06.008 25149893

[40] Micha RPJ Cudhea F Imamura F Rehm CD Mozaffarian D. Association between dietary factors and mortality from heart dis-ease, stroke, and type 2 diabetes in the United States. JAMA. (2017) 317:912–24. 10.1001/jama.2017.0947 28267855 PMC5852674

[41] Rees K Hartley L Flowers N Clarke A Hooper L Thorogood M. 'Mediterranean' dietary pattern for the primary prevention of cardiovascular disease. Cochrane Database Syst Rev. (2013) 2013:Cd009825. 10.1002/14651858.CD009825.pub2 23939686

[42] De Pergola G D'Alessandro A. Influence of mediterranean diet on blood pressure. Nutrients. (2018) 10:1700. 10.3390/nu10111700 30405063 PMC6266047

[43] Scicchitano P Cameli M Maiello M Modesti PA Muiesan ML Novo S. Nutraceuticals and dyslipidaemia: beyond the common therapeutics. J Funct Foods. (2014) 6:11–32. 10.1016/j.jff.2013.12.006

[44] Izzo R de Simone G Giudice R Chinali M Trimarco V De Luca N. Effects of nutraceuticals on prevalence of metabolic syndrome and on calculated Framingham risk score in individuals with dyslipidaemia. J Hypertens. (2010) 28:1482–7. 10.1097/HJH.0b013e3283395208 20498621

[45] Gunathilake KDPP Wang Y Vasantha Rupasinghea HP. Hypocholesterolemic and hypotensive effects of a fruitbased functional beverage in spontaneously hypertensive rats fed with cholesterol-rich diet. J Funct Foods. (2013) 5:1392–401. 10.1016/j.jff.2013.05.007

[46] Ozemek C Laddu DR Arena R Lavie CJ. The role of diet for prevention and management of hypertension. Curr Opin Cardiol. (2018) 33:388–93. 10.1097/HCO.0000000000000532 29771736

[47] Tsuchihashi T. Practical and personal education of dietary therapy in hypertensive patients. Hypertens Res. (2019) 43:6–12. 10.1038/s41440-019-0340-5 31576021

[48] Hecht EM Layton MR Abrams GA Rabil AM Landy DC. Healthy behavior adherence: the national health and nutrition examination survey, 2005-2016. Am J Prevent Med. (2020) 59:270–3. 10.1016/j.amepre.2020.02.013 32340777

[49] Dunn CG Wilcox S Saunders RP Kaczynski AT Blake CE Turner-McGrievy GM. Healthy eating and physical activity interventions in faith-based settings: a systematic review using the reach, effectiveness/efficacy, adoption, implementation, maintenance framework. Am J Prevent Med. (2021) 60:127–35. 10.1016/j.amepre.2020.05.014 33341177

[50] Martinez-Gonzalez MA Salas-Salvado J Estruch R Corella D Fito M Ros E. Benefits of the Mediterranean diet: insights from the PREDIMED study. Prog Cardiovasc Dis. (2015) 58:50–60. 10.1016/j.pcad.2015.04.003 25940230

[51] Selçuk KT Çevik C Mercan Y Koca H. Hypertensive patients' adherence to pharmacological and non-pharmacological treatment methods, in Turkey. IJCMPH. (2017) 4:2648–57. 10.18203/2394-6040.ijcmph20173308

[52] Maharjan B. Prevalence and awareness of hypertension among adults and its related risk factors. J Nepal Health Res Counc. (2018) 15:242–6. 10.3126/jnhrc.v15i3.18848 29353896

[53] Aaron KJ Campbell RC Judd SE Sanders PW Muntner P. Association of dietary sodium and potassium intakes with albuminuria in normal-weight, overweight, and obese participants in the Reasons for Geographic and Racial Differences in Stroke (REGARDS) Study. Am J Clin Nutr. (2011) 94:1071–8. 10.3945/ajcn.111.013094 21880845 PMC3173025

[54] He F MacGregor G. A comprehensive review on salt and health and current experience of worldwide salt reduction programmes. J Hum Hypertens. (2008) 23:363–84. 10.1038/jhh.2008.144 19110538

